# Crossing Vessel in Pelvi Ureteric Junction Obstruction: A Histopathological Analysis

**DOI:** 10.5152/tud.2022.22012

**Published:** 2022-07-01

**Authors:** Sanjeet Kumar Singh, Anjana Singh, Krishna Kumar Yadav, Gurunam Girniwale, Nuzhat Husain, Alok Srivastava, Chandra Kant Munjewar

**Affiliations:** 1 Department of Urology and Renal Transplantation, Dr Ram Manohar Lohia Institute of Medical Sciences, Lucknow, Uttar Pradesh, India; 2 Department of Pathology, Mayo Institute of Medical Sciences, Gadia, Barabanki, Uttar Pradesh, India; 3 Department of Pediatrics, Dr Ram Manohar Lohia Institute of Medical Sciences, Lucknow, Uttar Pradesh, India

**Keywords:** Pelvi ureteric junction obstruction, histopathology, laparoscopic pyeloplasty, crossing vessel

## Abstract

**Objective:**

The aim of the study is to identify whether crossing vessel is a cause or an associated finding in Pelvi Ureteric Junction Obstruction.

**Material and methods:**

This is a prospective study of a total of 128 patients who underwent laparoscopic pyeloplasty from January 2016 to June 2020. All patients who underwent laparoscopic pyeloplasty and pelvi ureteric junction segments were sent for histopathological examination. The presence of crossing vessels is documented intraoperative and patients were divided into two groups, group 1 having pelvi ureteric junction obstruction with crossing vessel, and group 2, pelvi ureteric junction obstruction without crossing vessels. Histopathological examination findings of pelvi ureteric junction segment including inflammation, fibrosis, muscle hypertrophy, muscle disarray, and synaptophysin were recorded. Unpaired Student *t*-test was used for comparing differences between continuous normally distributed data from 2 samples and non-parametric tests were applied for continuous data.

**Results:**

Of the total 128 patients, crossing vessels were identified in 42 (32.8%), and 86 (67.2%) were without crossing vessels. The demographic profile of patients between the 2 groups was comparable. On histopathological examination, moderate-to-severe chronic inflammation was seen in 23.8% and 44.2% (*P* > .05) in group 1 and group 2, respectively; fibrosis and muscular hypertrophy were higher in group 2 but statistically insignificant (*P* > .05), and muscle disarray was higher in group 1 but statistically insignificant (*P* > .05). Synaptophysin was positive in 4.8% and 4.7% in group 1 and group 2, respectively.

**Conclusion:**

The differences in histopathological examination between the 2 groups were not statistically significant. However, in patients with crossing vessels, there was a higher degree of inflammation, which may lead to early pelvi ureteric junction obstruction.

**Main Points**

- To assess whether crossing vessel is a cause or associated finding of pelvi ureteric junction obstruction, a histopathological examination of pelvi ureteric junction segment was done in patients with and without crossing vessel. Histopathological examination findings of pelvi ureteric junction segment include inflammation, fibrosis, muscle hypertrophy, muscle disarray, and synaptophysin.
- On histopathological examination, moderate-to-severe chronic inflammation was higher in patients with crossing vessels and fibrosis and muscular hypertrophy were higher in patients without crossing vessels, but these differences were statistically insignificant.
- Similarly, muscle disarray was statistically insignificant in patients with crossing vessels. Synaptophysin was positive in both groups.
- Since histopathological examination findings were statistically insignificant in the patients with and without crossing vessels, it can be concluded that crossing vessel is not a cause of pelvi ureteric junction obstruction rather it is an associated finding.
- Further studies using electron microscopy along with histopathology will be required to see the degree of different components responsible for pelvi ureteric junction obstruction. This may help in deciding whether pyeloplasty should be done or go for transposition of crossing vessels only without pyeloplasty.

## Introduction

Pelvic ureteric junction obstruction (PUJO) is more common in children. It is twice as prevalent in males as it is in females, and it is twice as common on the left side as it is on the right.^1^ Its estimated global incidence is 1 in 1500.^1^ The most prevalent cause is assumed to be an intrinsic abnormality of muscle development or insufficient nerves in the obstructed PUJ segment.^2^ Other causes are (i) obstruction from extrinsic compression such as crossing vessel (CV), tumor compression, fibrous cord, enlarged lymph node, etc.; (ii) intramural pathology (fibrosis of PUJ segment secondary to previous surgery, stones, or tumor); (iii) intraluminal pathology (stones, polyp, mucosal folds, etc.). The role of renal CV in patients with PUJO has been the subject of debate.^3-5^ Although the real frequency of CV in the normal unobstructed PUJ population is unknown, studies have found that it ranges from 39% to 65%.^6,7^

Crossing vessel is not the primary cause of PUJO rather it is already obstructed due to intrinsic defect and it only causes partial obstruction leading to redundant pelvis kinks and falls upon increasing the vessel hydronephrosis.^8^ Some authors assumed that this vessel was the sole source of the obstruction, and its transposition only relieved the obstruction.^9^

Despite this link, the definitive causal relationship between CV and PUJO, if there is one, is unknown. The purpose of this study was to evaluate histopathological changes in PUJ segment in patients of PUJO with or without CV and whether the CV is the cause of obstruction or an associated finding.

## Material and Methods

This is a prospective observational study done from January 2016 to June 2020. Patients with hydronephrosis on ultrasound had their PUJO confirmed by a diethylenetriamine pentaacetate (DTPA) scan. Patients having obstructed patterns on DTPA scan were taken for the study. All underwent laparoscopic dismembered Anderson Hynes (AH) pyeloplasty. The presence of CV was noted during surgery. After surgery, the PUJ segment was sent for histopathological examination (HPE). Patients were divided into 2 groups: group 1, PUJO with CV, and group 2, PUJO without CV. A total of 42 patients were included in group 1 and 86 patients in group 2. Data include demographic profile, laterality, presence of CV, and HE of PUJ segment including inflammation, fibrosis, muscle hypertrophy, muscle disarray, and synaptophysin.

The data were collected on a predesigned schedule and subsequently entered in Microsoft Excel®. The proportions were presented as percentages and continuous data were presented as mean ± SD. Unpaired Student *t*-test was used for comparing differences between continuous normally distributed data from 2 samples. The proportions were analyzed using chi-squared tests. A *P*-value of less than .05 was considered significant.

Prior to the recruitment of the study’s participants, written informed consent was obtained. Patients, who refused to participate in the trial, as well as those with secondary PUJO and recurrent PUJO, were excluded. Prior to the start of subject recruiting, institutional ethics and review board approval were obtained from Dr. R. M. L. Institute of Medical Sciences, Lucknow, India (IEC No: 40/17).

### Surgical Assessment

All of the patients who had PUJO were treated with laparoscopic trans-peritoneal AH pyeloplasty. The pre-operative finding of CV was noted. Crossing vessels consist of an artery and/or a vein. The artery originated from aorta and vein drain to inferior vena cava. It is usually present in relation to inferior pole of the kidney. Crossing vessel was transposed posteriorly. The redundant pelvis, PUJ, and a narrow segment of the ureter below the PUJ were removed and preserved in a 10% formalin solution. This specimen was sent for HPE.

### Histopathological Examination

Pelvic ureteric junction segment was assessed for inflammation, fibrosis, muscle hypertrophy, pattern of smooth muscle, and synaptophysin. All PUJ segments were fixed in 10% (wt/vol) phosphate-buffered in formalin for 24–48 h. After doing standard histological processing and embedding in paraffin, 5-µm-thick sections were used for H&E staining. These stained sections were examined for the presence of inflammation, fibrosis, muscle hypertrophy, and muscle disarray. Immunohistochemistry was done for synaptophysin. Histopathologist was blinded to the presence or absence of CV. Urothelium was evaluated for the presence or absence of metaplasia/dysplasia. In lamina propria layer, presence of fibrosis (0 = none; mild = limited to lamina propria; moderate = involving muscularis propria; severe = replacing muscularis propria and extending into adventitial layer) and inflammation (divided into mild, moderate, and severe as above) was evaluated.^10^

A special stain, Masson’s trichrome, was used to differentiate between fibrosis and smooth muscle hyperplasia. It was also used for defining muscle disarray. Synaptophysin staining was done to look for ganglion cells in the wall.

## Results

None of the patients required conversion to open pyeloplasty after undergoing laparoscopic Anderson Hynes (AH) pyeloplasty. The incidence of CV in individuals with PUJO was 32.8% (n = 42). The demographic findings of the patients are given in Table 1.

**Figure 1.**
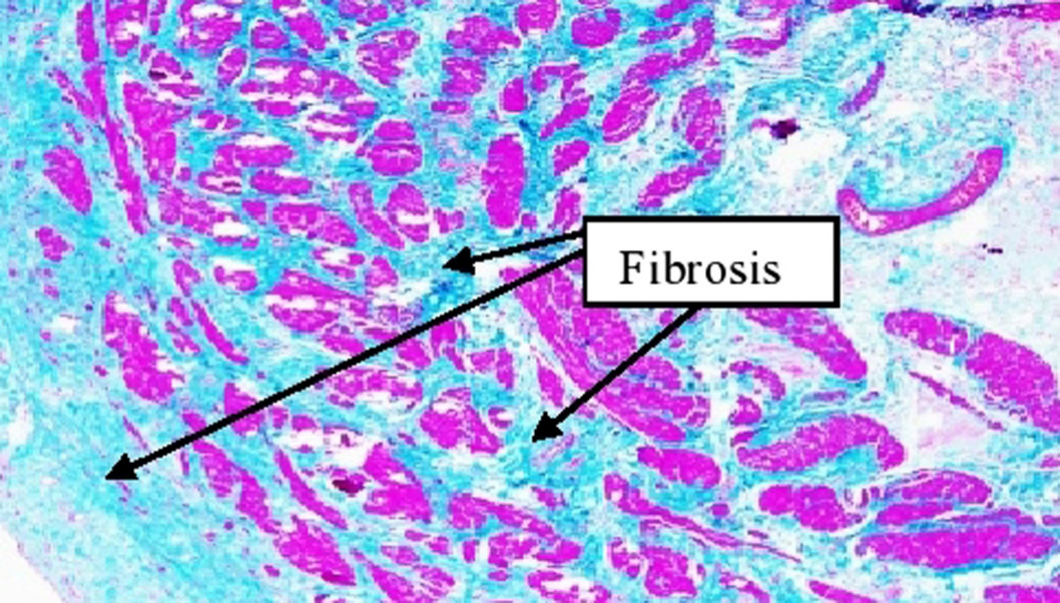
Fibrosis grade 2 of PUJ segment in group 1. PUJ, pelvi ureteric junction.

**Figure 2.**
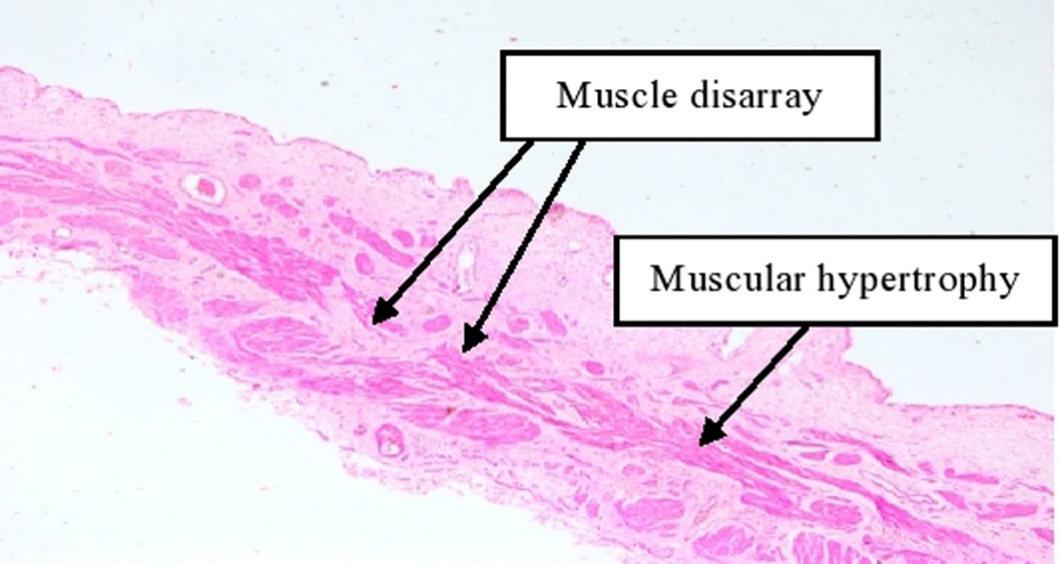
Fibrosis grade 1, mild muscle hypertrophy, absent muscle disarray of PUJ segment in group 2. PUJ, pelvi ureteric junction.

There are no statistically significant differences in the HPE findings including inflammation, fibrosis, muscle hypertrophy, muscular disarray, and synaptophysin between these two groups. The HPE findings were not observed to be affected by age. The HPE findings are given in Table 2. Histopathological features are shown in Figures 1 and 2.

## Discussion

The pathophysiology of PUJO is unknown. The decrease in distensability at the obstructed segments might be explained by a change in muscle configuration, atrophy/drop in myocytes, decrease in Cajal interstitial cells, reduction of nerve terminals, and increased collagen deposition between muscle bundles. These may contribute to the absence of the ureteropelvic muscular contractions of PUJ segment.^2,11-13^

On the other hand, few researchers opine that an intrinsic abnormality in muscle cells is the cause of obstruction and that morphological changes are secondary.^14^ The involvement of CV in the pathophysiology and histological alterations of the PUJ segment in PUJO patients is unknown. If ureter compression and obstruction caused by CV were the primary cause of PUJO, the HPE of this segment in those with and without CV should differ. Incidence of CV in literature ranges from 19.8% to 63%.^2,15,16^ In the present study, CV was found in 32.8% (n = 42). 

Researchers like Cancian et al^10^ Dogan et al^16^ and Ellerkamp et al^17^ also found similar results. Dogan et al^16^ compared patients of PUJO without CV (n = 57), with CV (n = 17), and normal PUJ (n = 12). All cases were compared for number of interstitial cells muscle layer, number of neurons at the level of lamina propria, and presence or absence of fibrosis/inflammation. They did not find statistically significant differences. Similarly, Ellerkamp et al^17^ and Cancian et al^10^ graded fibrosis, muscle hypertrophy, and inflammation and demonstrated no differences between these two groups.

Murakumo et al^2^ examined histopathologically along with electron microscopy of PUJ segment of healthy (n = 7), with CV (n = 4), and without CV (n = 7). The study showed decreased nerve fibers, atrophy of muscle fibers, and increased collage deposits in patients without CV. However, there was no discernible change in the presence of synaptophysin in patients with and without CV. This could be related to the use of electron microscopy to examine nerve tissue and the small number of participants in the study.^2^

Contrary to present study, Lee Richstone et al^15^ studied 65 patients with CV and 30 patients without CV and found significant difference in histopathological changes of chronic inflammation, fibrosis, muscle hypertrophy, and muscle atrophy.^15^ Since this was a retrospective study based on simple H&E stained PUJ segment for chronic inflammation, fibrosis, muscle hypertrophy, and muscular atrophy, it could be difficult to identify neurological or molecular pathogenic characteristics. In the present study, a special stain, Masson’s trichrome was used to differentiate fibrosis from muscle hypertrophy. They also did not consider the grades of inflammation, fibrosis, and muscle hypertrophy rather noted the presence or absence of these. Presence of synaptophysin was detected by immune-histochemistry in the present study. 

On the other hand, just transposing of CV (using Hellstrom procedure) alleviates the obstruction.^18,19^ This procedure can be done in PUJO with visible peristalsis in PUJ segment, apparently healthy ureter and after vascular displacement, diuretic-response with emptying of the dilated pelvis to confirm blockage release and rule out intrinsic-PUJ abnormalities.^20^

The presence of CV causes increased inflammation in the PUJ segment. This inflammation causes adhesion to nearby structures, causing obstruction.^10^ The degree or proportion of different components at PUJ was not studied. It is unknown at what percent these components cause significant blockage at the PUJ. There is dearth of studies based on HPE of PUJ segment in PUJO. The strength of our study is that we studied a larger number of patients along with studying for all components of tissue elements leading to fibrosis and obstruction. The limitation of the present study is that we did not see the degree of these components at what proportion they lead to significant obstruction at PUJ segment. 

Histopathological examination of PUJ was same in both groups. It is difficult to conclude that CV is a cause or associated structure depending on the findings of the present study. Therefore, further study based on not only histopathology but also including electron microscopy should be considered. Additionally, since peristalsis is triggered and controlled by Cajal cells and the level of Caveolin-1 should be taken into account in the further studies, this may help in deciding whether we do pyeloplasty or go for transposition of CV only without pyeloplasty.

In conclusion, the differences in HPE between the two groups were not statistically significant. However, in patients with CV, there was a higher degree of inflammation, which may lead to early PUJ obstruction. 

## Figures and Tables

**Table 1.** Demographic Profile

**Characteristic **	Group 1 (with CV) n = 42 (32.8%)	Group 2 (without CV) n = 86 (67.2%)	*P*
Age (mean ± SD) (years)	29.57 ± 10.50	30.05 ± 9.94	.861
Sex (male/female)	30/12	54/32	.495
Laterality(left/right)	14/28	40/46	.326
Pre-operatvie split function (mean ± SD)	29.69 ± 6.70	31.28 ± 6.29	.191
Antero-posterior pelvic diameter (cm)	7.14 ± 1.85	6.9 ± 1.95	.507

CV, crossing vessel.

**Table 2.** Findings of Histopathological Examination (HPE)

**Characteristics**	Grade	Group 1 (with CV)		Group 2 (without CV)		95% CI	*P*
n	%	n	%
Inflammation	M	16	76.2	24	55.8	−0.73,0.277	.114
M–S	5	23.8	19	44.2	−0.233,0.03
Fibrosis	M	6	28.6	10	23.3	−0.099,0.152	.645
M–S	15	71.4	33	76.7	−0.204,0.151
Synaptophysin	Positive	1	4.8	2	4.7	−0.055,0.057	1.000
Negative	20	95.2	41	95.3	−0.185,0.184
Muscle hypertrophy	M	11	52.4	30	69.8	−0.254,0.08	.173
M–S	10	47.6	13	30.2	−0.062,0.236
Muscle disarray		5	23.8	3	7.0	−0.021,0.19	.102

CV, crossing vessel.

## References

[1] Grasso M Caruso RP Phillips CK . UPJ obstruction in the adult population: are crossing vessels significant? Rev Urol. 2001;3(1):42 51. 16985690 PMC1476031

[2] Murakumo M Nonomura K Yamashita T Ushiki T Abe K Koyanagi T . Structural changes of collagen components and diminution of nerves in congenital ureteropelvic junction obstruction. J Urol. 1997;157(5):1963 1968. 10.1016/S0022-5347(01)64910-3) 9112572

[3] Sampaio FJ The dilemma of the crossing vessel at the ureteropelvic junction: precise anatomic study. J Endourol. 1996;10(5):411 415. 10.1089/end.1996.10.411) 8905485

[4] Sampaio FJ Vascular anatomy at the ureteropelvic junction. Urol Clin North Am. 1998;25(2):251 258. 10.1016/s0094-0143(05)70012-4) 9633579

[5] Zeltser IS Liu JB Bagley DH . The incidence of crossing vessels in patients with normal ureteropelvic junction examined with endoluminal ultrasound. J Urol. 2004;172(6 Pt 1):2304 2307. 10.1097/01.ju.0000145532.48711.f6) 15538254

[6] Sampaio FJB Ureteropelvic junction anatomy. Atlas Urol Clin. 2003;11(2):129 140. 10.1016/S1063-5777(03)00042-2)

[7] Van Cangh PJ Wilmart JF Opsomer RJ Abi-Aad A Wese FX Lorge F . Long term results and late recurrence after endopyelotomy:a critical analysis of prognostic factors. J Urol. 1994;151(4):934-937. 10.1016/s0022-5347(17)35126-1) 8126829

[8] De Siati M Silvestre P Scieri F Breda G . Congenital ureteropelvic junction obstruction:definition and therapy. Arch Ital Urol Androl. 2005;77(1):1 4. 15906780

[9] Sakoda A Cherian A Mushtaq I . Laparoscopic transposition of lower pole crossing vessels (‘vascular hitch’) in pure extrinsic pelviureteric junction (PUJ) obstruction in children. BJU Int. 2011;108(8):1364 1368. 10.1111/j.1464-410X.2011.10657.x) 21958225

[10] Cancian M Pareek G Caldamone A Aguiar L Wang H Amin A . Histopathology in ureteropelvic junction obstruction With and Without crossing vessels. Urology. 2017;107:209 213. 10.1016/j.urology.2017.05.013) 28526243

[11] Solari V Piotrowska AP Puri P . Altered expression of interstitial cells of Cajal in congenital ureteropelvic junction obstruction. J Urol. 2003;170(6 Pt 1):2420 2422. 10.1097/01.ju.0000097401.03293.f0) 14634443

[12] Hanna MK Jeffs RD Sturgess JM Barkin M . Ureteral structure and ultrastructure. Part II. Congenital ureteropelvic junction obstruction and primary obstructive megaureter. J Urol. 1976;116(6):725 730. 10.1016/s0022-5347(17)58987-9) 1003640

[13] Seremetis GM Maizels M . TGF-beta mRNA expression in the renal pelvis after experimental and clinical ureteropelvic junction obstruction. J Urol. 1996;156(1):261 266. 10.1016/S0022-5347(01)66013-0) 8648819

[14] Gosling JA Dixon JS . Functional obstruction of the ureter and renal pelvis. A histological and electron microscopic study. Br J Urol. 1978;50(3):145 152. 10.1111/j.1464-410x.1978.tb02790.x) 753449

[15] Richstone L Seideman CA Reggio E et al. Pathologic findings in patients with ureteropelvic junction obstruction and crossing vessels. Urology. 2009;73(4):716 9; discussion 719. 10.1016/j.urology.2008.10.069) 19193425

[16] Doğan HT Canda AE Gök B et al. Is there a difference in the number of interstitial cells, neurons, presence of fibrosis and inflammation in ureteropelvic junction tissues of patients with ureteropelvic junction obstruction with and without crossing vessels? Turk J Urol. 2019;45(4):302 306. 10.5152/tud.2018.08784) 30201079 PMC6619840

[17] Ellerkamp V Kurth RR Schmid E Zundel S Warmann SW Fuchs J . Differences between intrinsic and extrinsic ureteropelvic junction obstruction related to crossing vessels: histology and functional analyses. World J Urol. 2016;34(4):577 583. 10.1007/s00345-015-1645-x) 26219514

[18] Godbole P Mushtaq I Wilcox DT Duffy PG . Laparoscopic transposition of lower pole vessels--the 'vascular hitch': an alternative to dismembered pyeloplasty for pelvi-ureteric junction obstruction in children. J Pediatr Urol. 2006;2(4):285 289. 10.1016/j.jpurol.2005.11.017) 18947622

[19] Madec FX Faraj S Villemagne T Fourcade L Lardy H Leclair MD . Laparoscopic transposition of lower-pole crossing vessels: long-term follow-up of 33 patients at puberty. J Pediatr Urol. 2016;12(4):226.e1 226.e6. 10.1016/j.jpurol.2016.03.016) 27238751

[20] Esposito C Bleve C Escolino M et al. Laparoscopic transposition of lower pole crossing vessels (vascular hitch) in children with pelviureteric junction obstruction. Transl Pediatr. 2016;5(4):256 261. 10.21037/tp.2016.09.08) 27867849 PMC5107373

